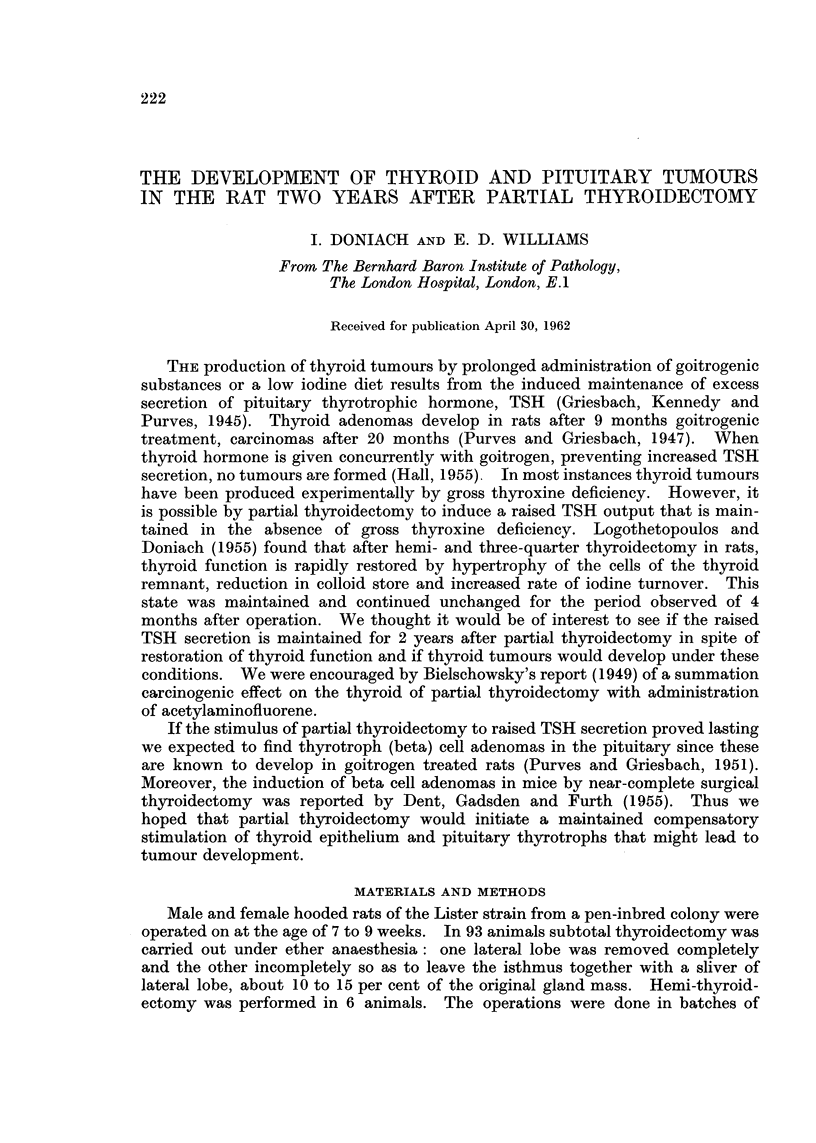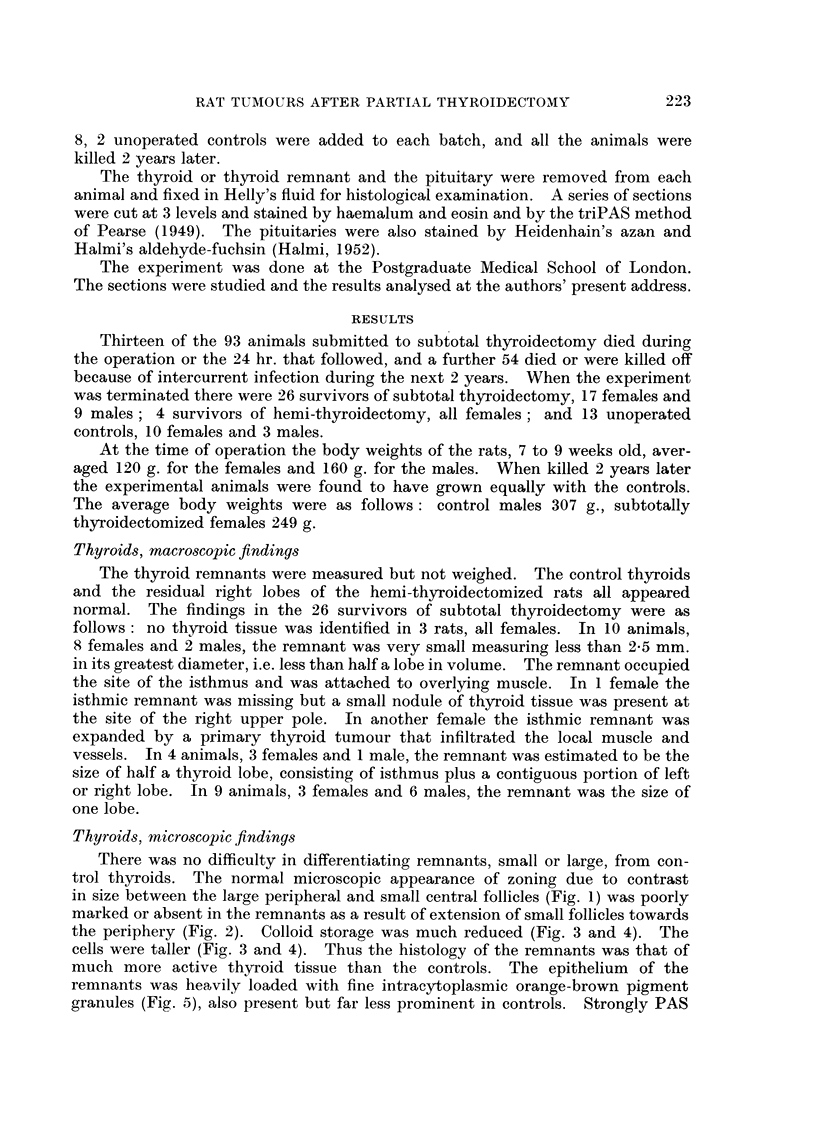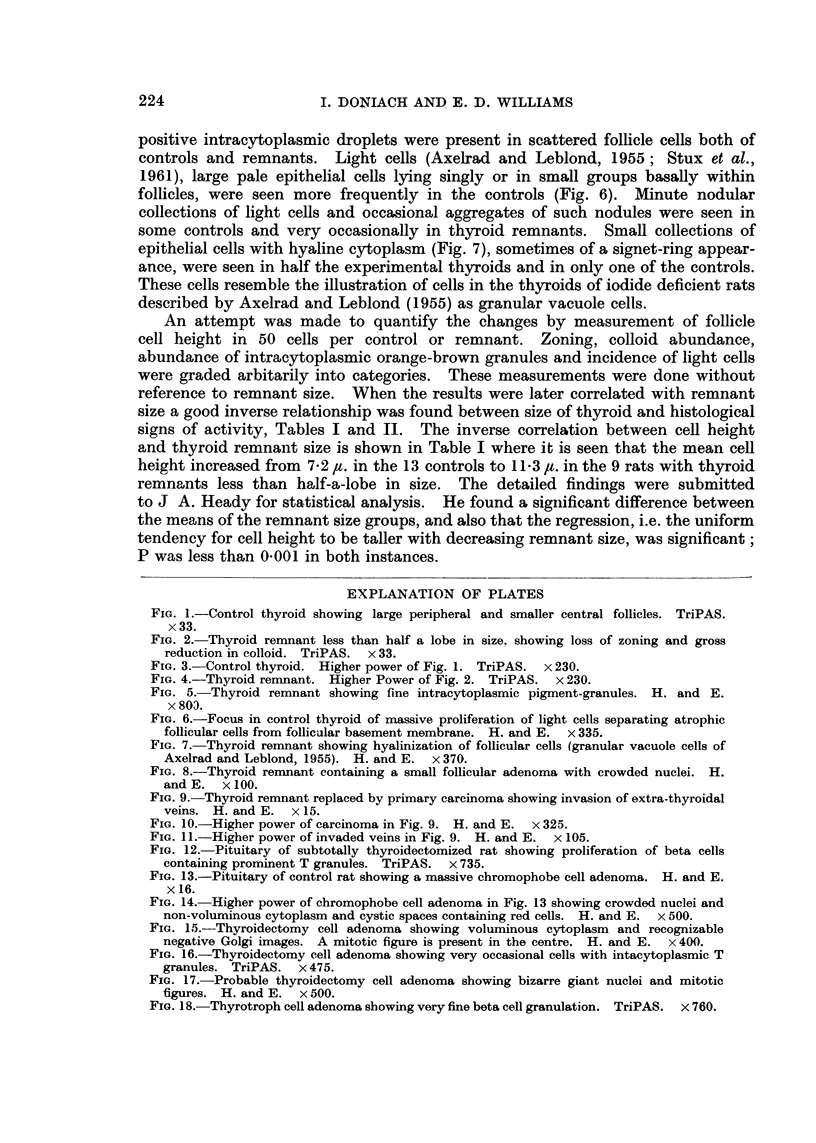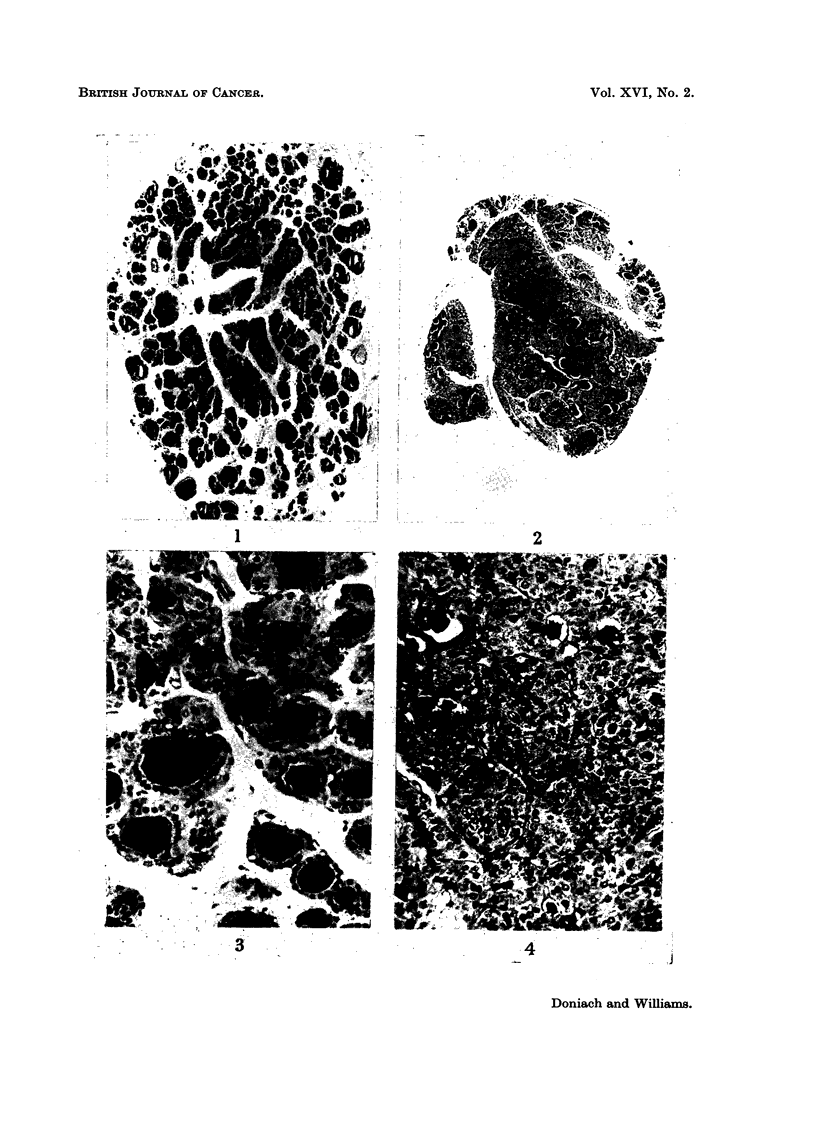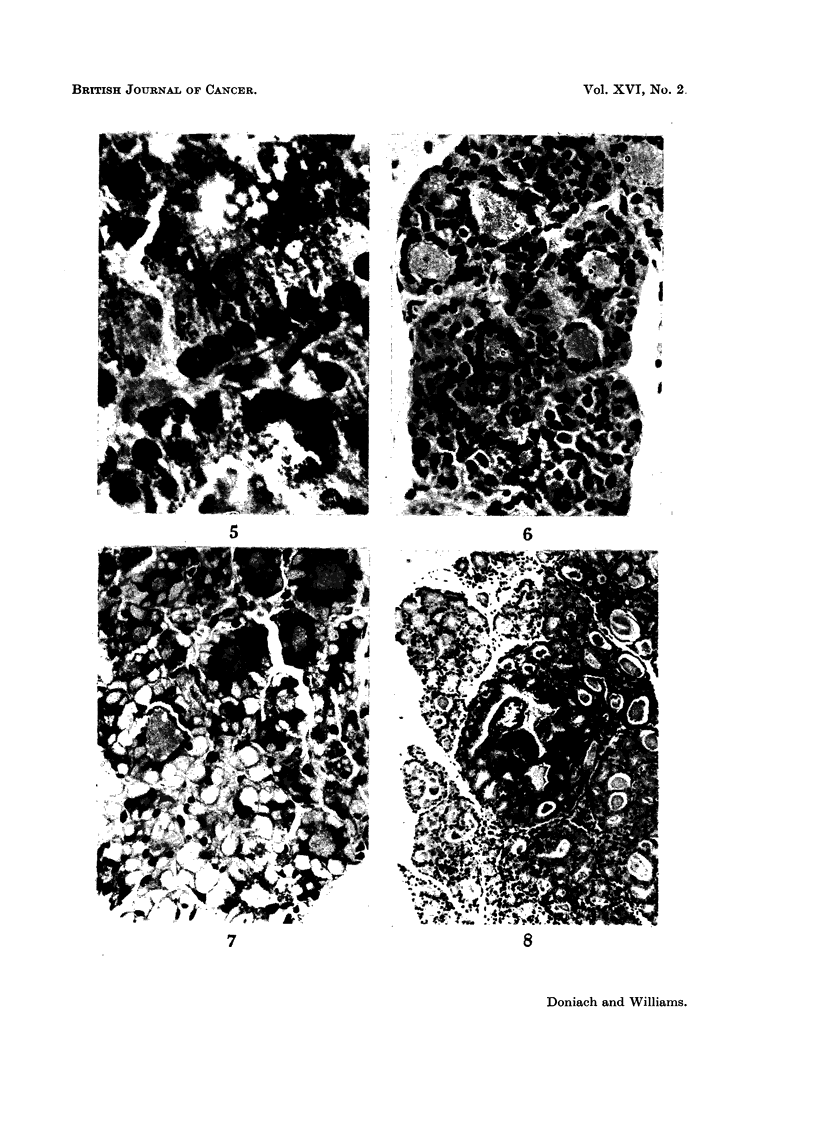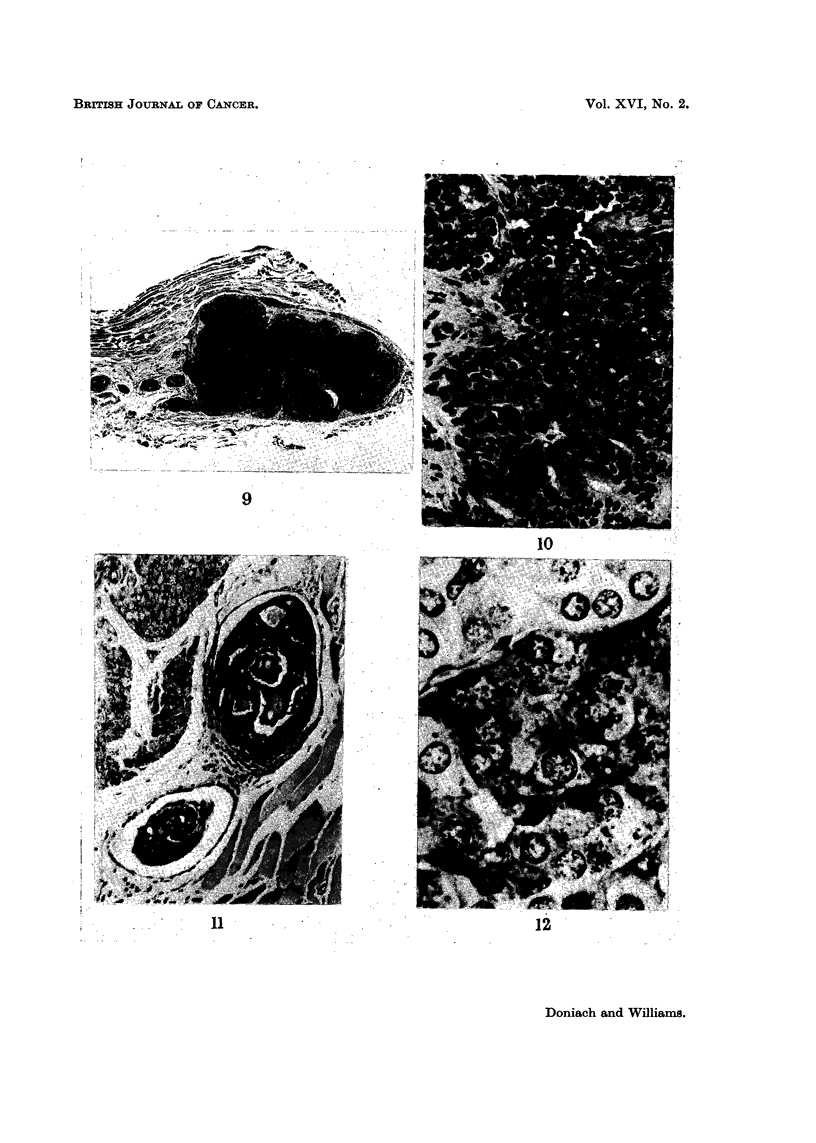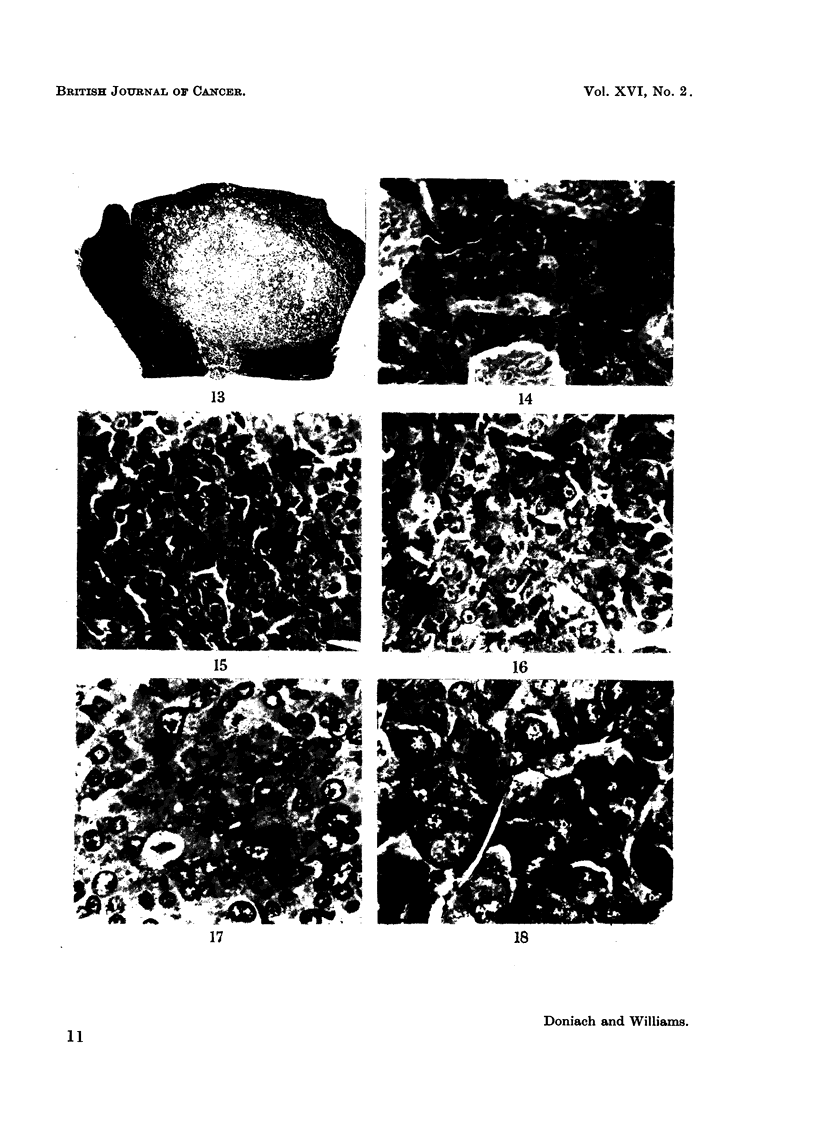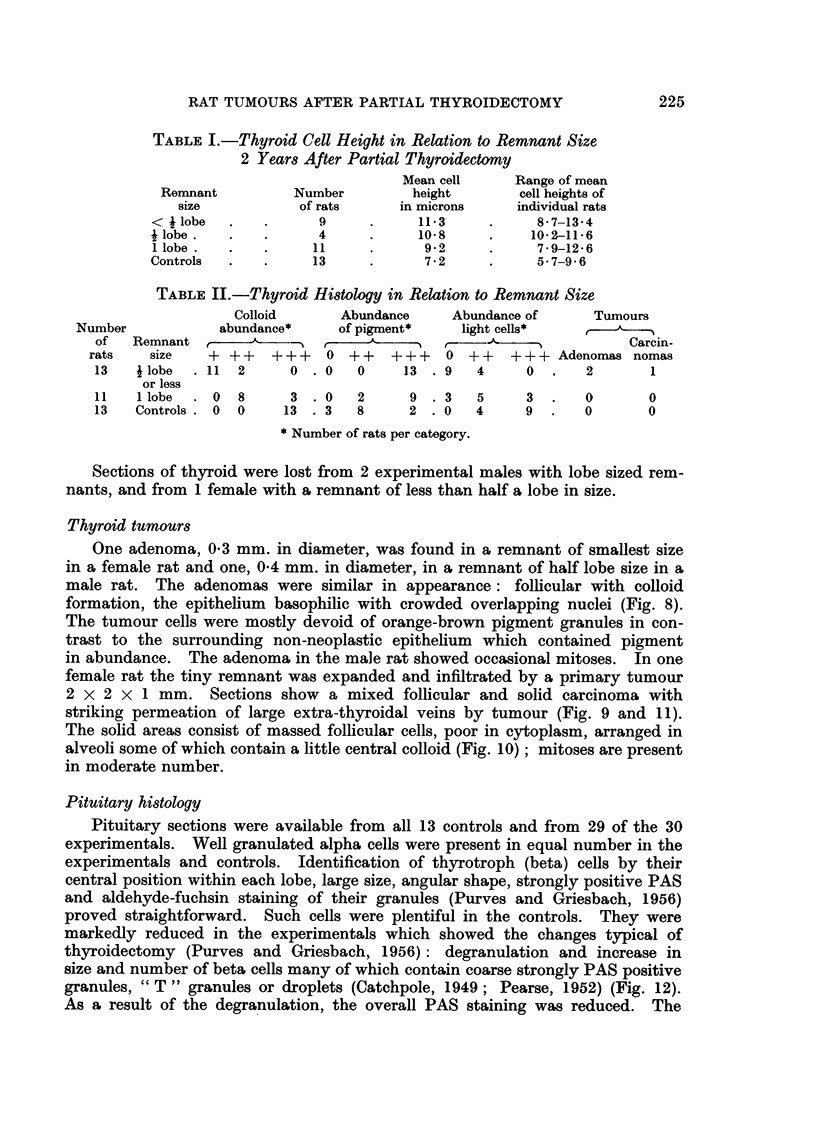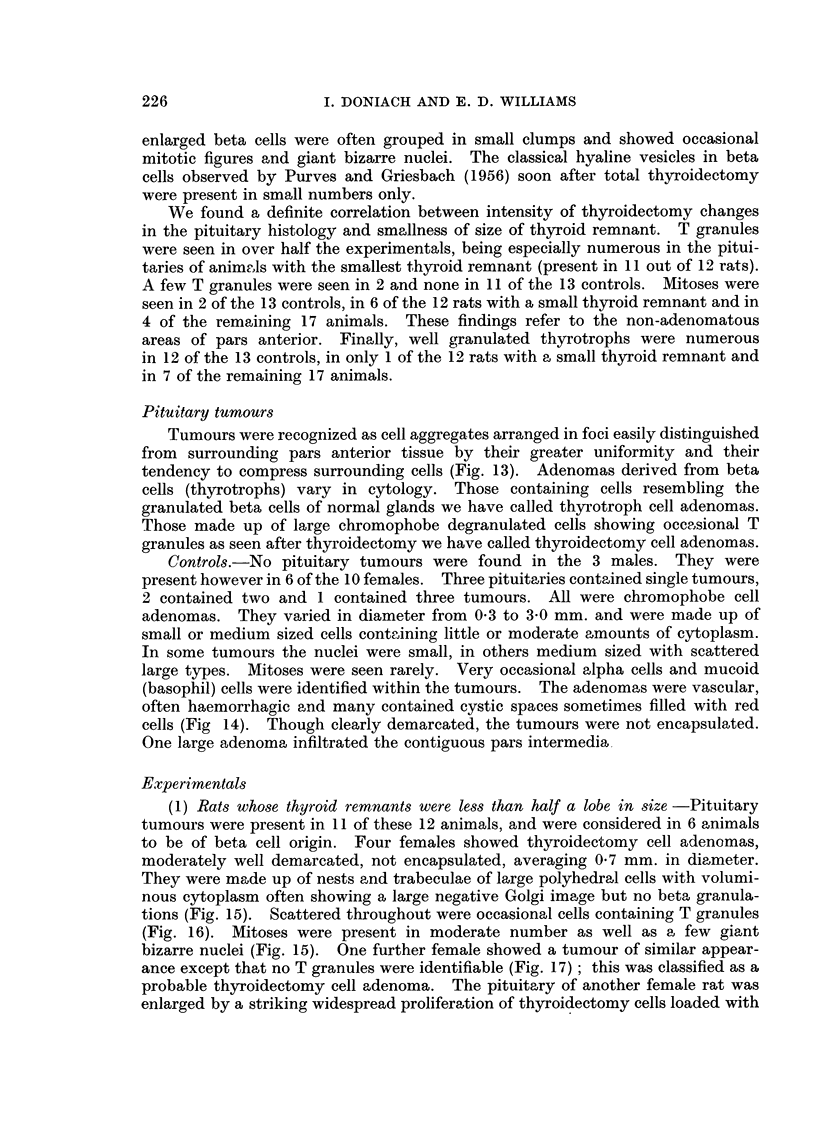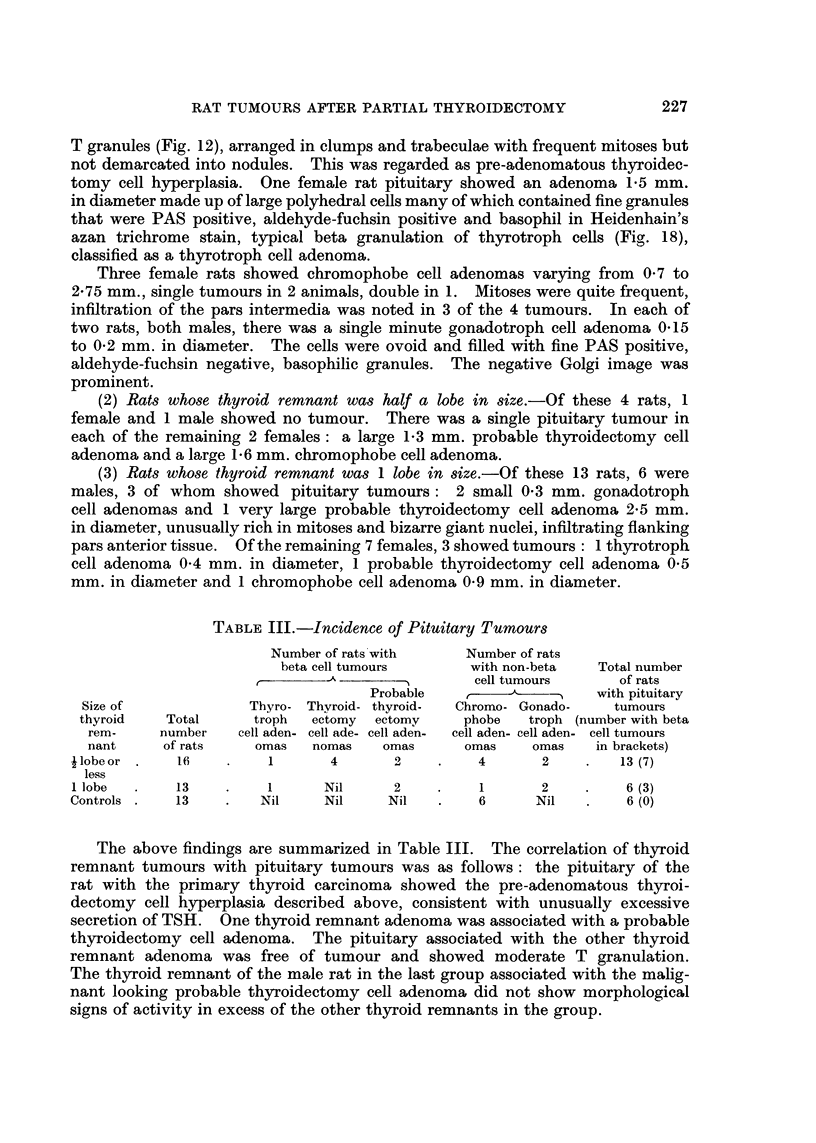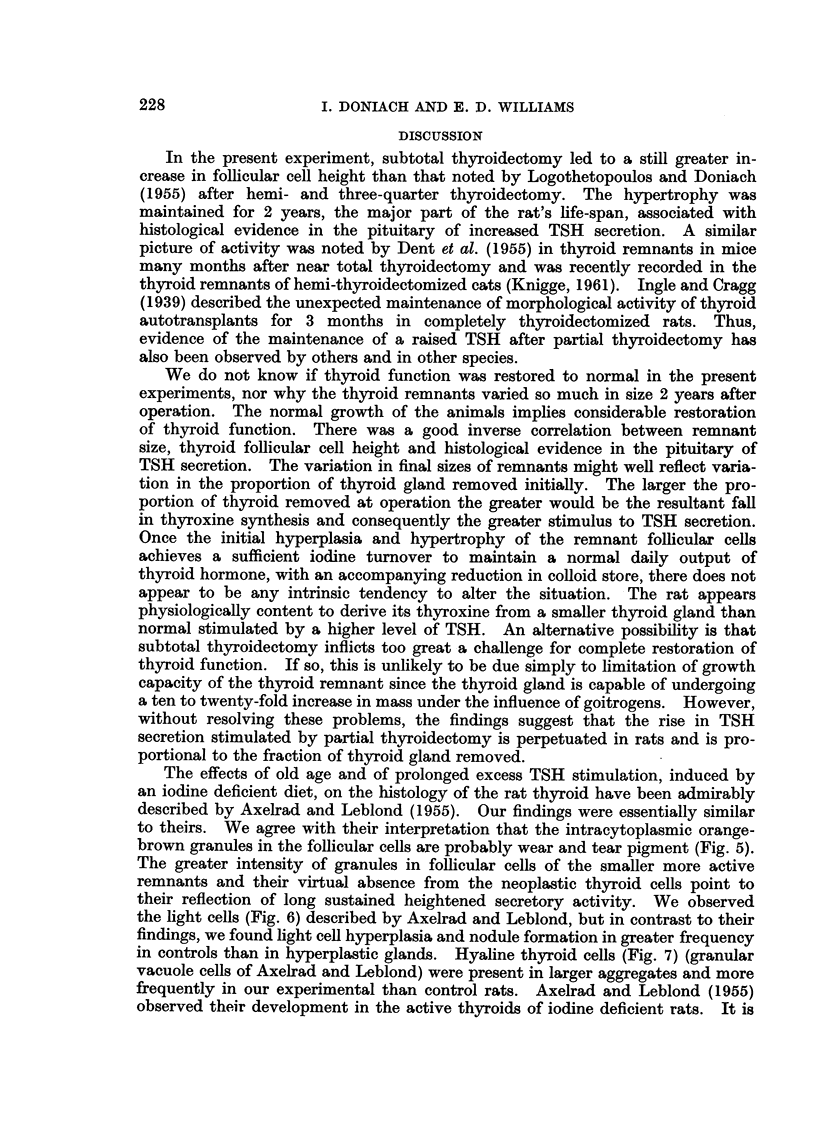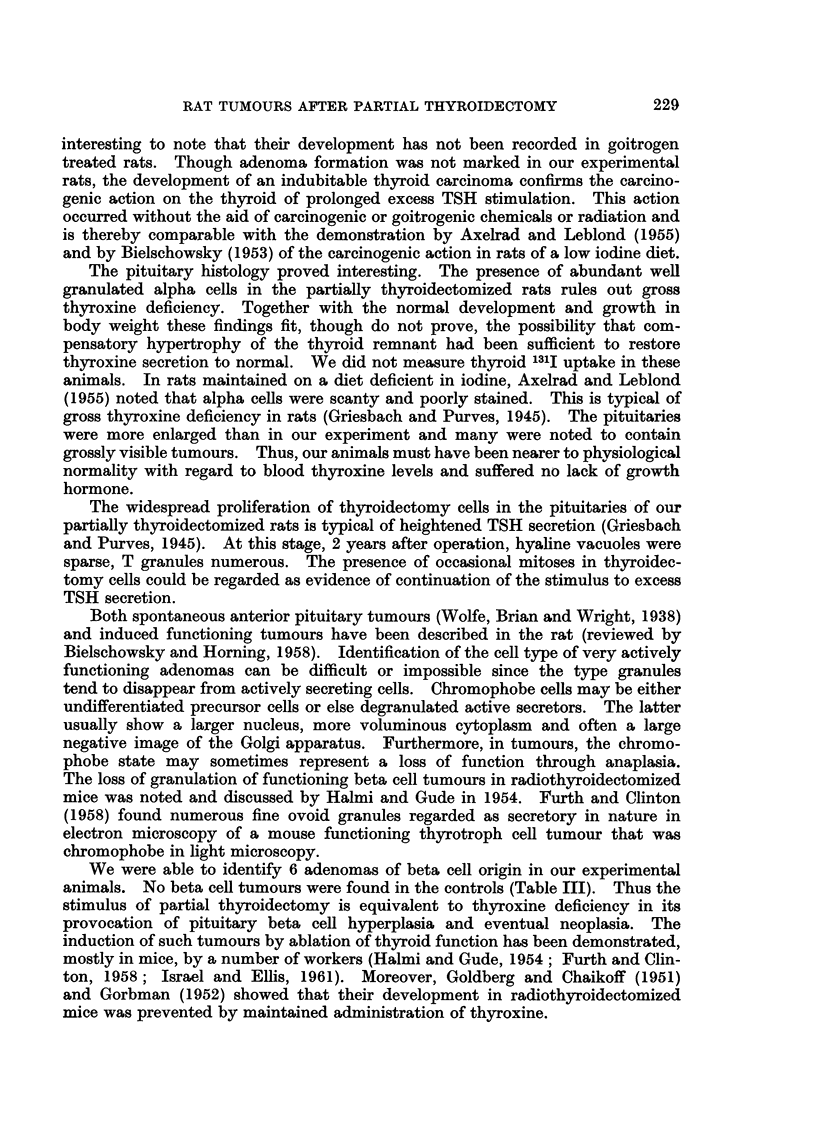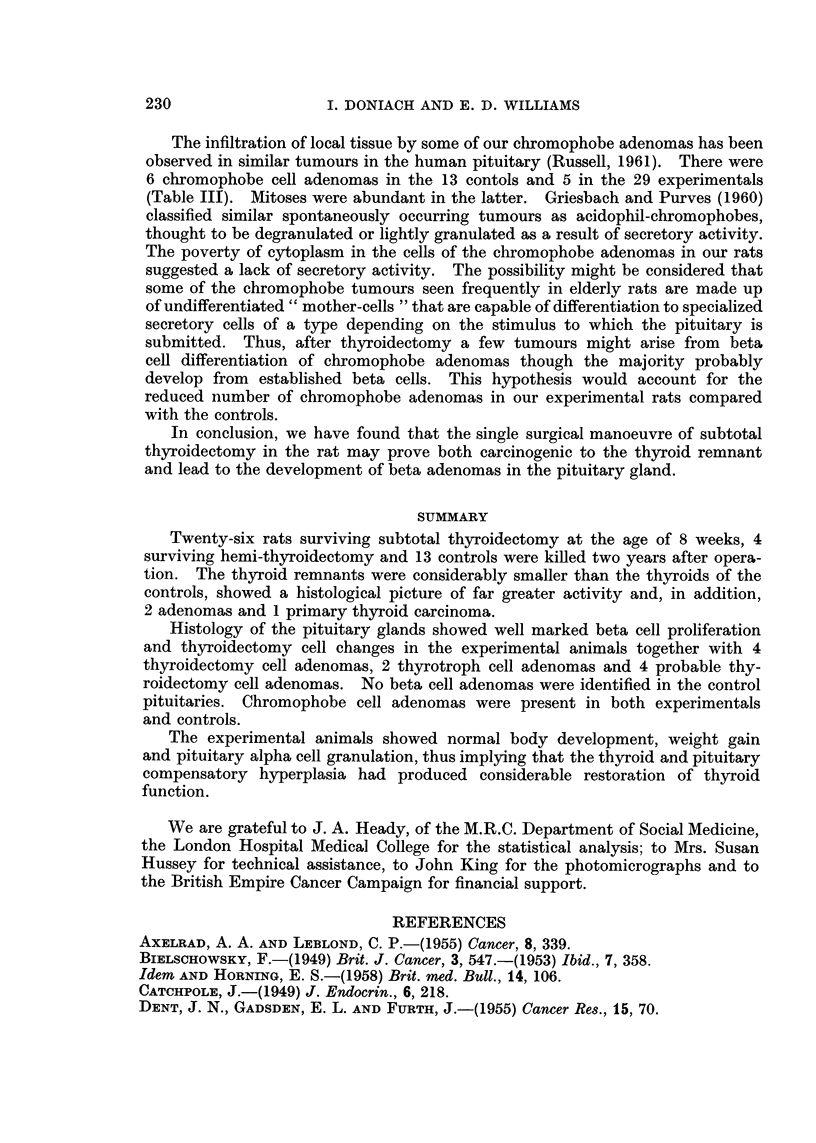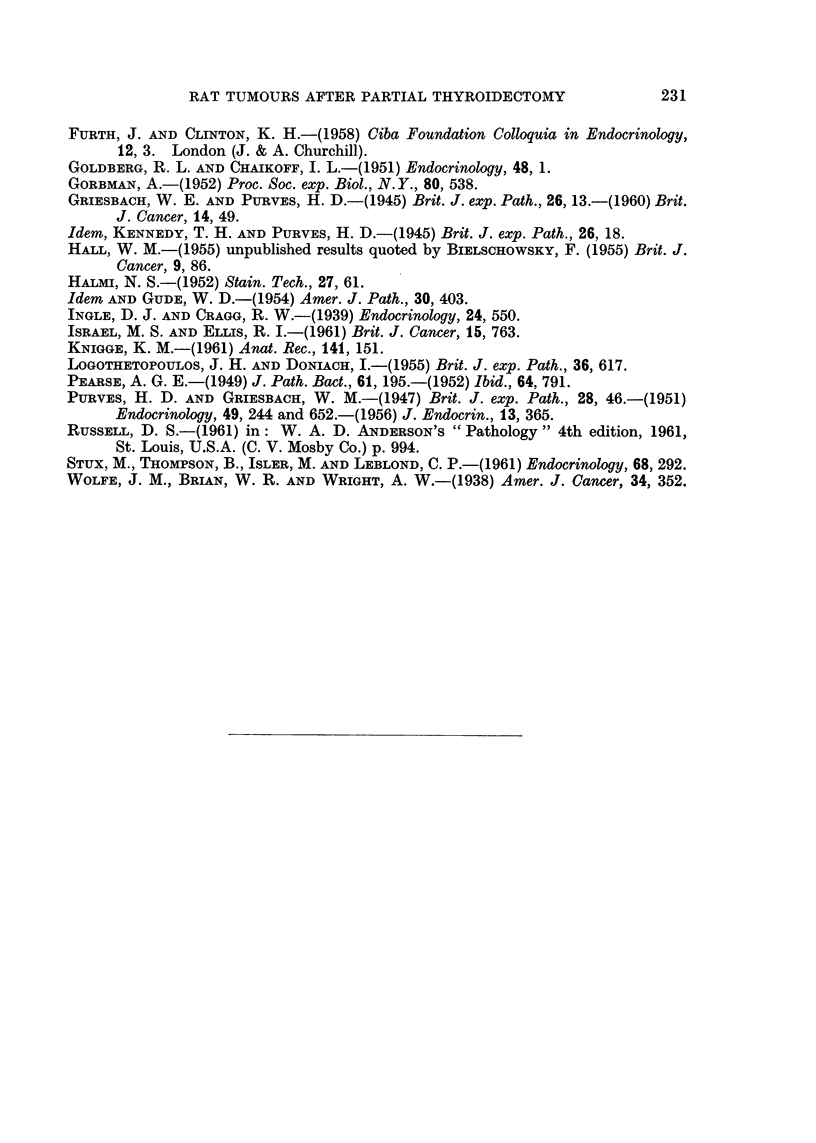# The Development of Thyroid and Pituitary Tumours in the Rat Two Years after Partial Thyroidectomy

**DOI:** 10.1038/bjc.1962.25

**Published:** 1962-06

**Authors:** I. Doniach, E. D. Williams

## Abstract

**Images:**


### THE DEVELOPMENT OF THYROID AND PITUITARY TUMOURS IN THE RAT TWO YEARS AFTER PARTIAL THYROIDECTOMY

I. DONIACH AND E. D. WILLIAMS

*From The Bernhard Baron Institute of Pathology, The London Hospital, London, E.1*

Received for publication April 30, 1962

THE production of thyroid tumours by prolonged administration of goitrogenic substances or a low iodine diet results from the induced maintenance of excess secretion of pituitary thyrotrophic hormone, TSH (Griesbach, Kennedy and Purves, 1945). Thyroid adenomas develop in rats after 9 months goitrogenic treatment, carcinomas after 20 months (Purves and Griesbach, 1947). When thyroid hormone is given concurrently with goitrogen, preventing increased TSH secretion, no tumours are formed (Hall, 1955). In most instances thyroid tumours have been produced experimentally by gross thyroxine deficiency. However, it is possible by partial thyroidectomy to induce a raised TSH output that is maintained in the absence of gross thyroxine deficiency. Logothetopoulos and Doniach (1955) found that after hemi- and three-quarter thyroidectomy in rats, thyroid function is rapidly restored by hypertrophy of the cells of the thyroid remnant, reduction in colloid store and increased rate of iodine turnover. This state was maintained and continued unchanged for the period observed of 4 months after operation. We thought it would be of interest to see if the raised TSH secretion is maintained for 2 years after partial thyroidectomy in spite of restoration of thyroid function and if thyroid tumours would develop under these conditions. We were encouraged by Bielschowsky's report (1949) of a summation carcinogenic effect on the thyroid of partial thyroidectomy with administration of acetylaminofluorene.

If the stimulus of partial thyroidectomy to raised TSH secretion proved lasting we expected to find thyrotroph (beta) cell adenomas in the pituitary since these are known to develop in goitrogen treated rats (Purves and Griesbach, 1951). Moreover, the induction of beta cell adenomas in mice by near-complete surgical thyroidectomy was reported by Dent, Gadsden and Furth (1955). Thus we hoped that partial thyroidectomy would initiate a maintained compensatory stimulation of thyroid epithelium and pituitary thyrotrophs that might lead to tumour development.

### MATERIALS AND METHODS

Male and female hooded rats of the Lister strain from a pen-inbred colony were operated on at the age of 7 to 9 weeks. In 93 animals subtotal thyroidectomy was carried out under ether anaesthesia : one lateral lobe was removed completely and the other incompletely so as to leave the isthmus together with a sliver of lateral lobe, about 10 to 15 per cent of the original gland mass. Hemi-thyroidectomy was performed in 6 animals. The operations were done in batches of

8, 2 unoperated controls were added to each batch, and all the animals were killed 2 years later.

The thyroid or thyroid remnant and the pituitary were removed from each animal and fixed in Helly's fluid for histological examination. A series of sections were cut at 3 levels and stained by haemalum and eosin and by the triPAS method of Pearse (1949). The pituitaries were also stained by Heidenhain's azan and Halmi's aldehyde-fuchsin (Halmi, 1952).

The experiment was done at the Postgraduate Medical School of London. The sections were studied and the results analysed at the authors' present address.

### RESULTS

Thirteen of the 93 animals submitted to subtotal thyroidectomy died during the operation or the 24 hr. that followed, and a further 54 died or were killed off because of intercurrent infection during the next 2 years. When the experiment was terminated there were 26 survivors of subtotal thyroidectomy, 17 females and 9 males ; 4 survivors of hemi-thyroidectomy, all females ; and 13 unoperated controls, 10 females and 3 males.

At the time of operation the body weights of the rats, 7 to 9 weeks old, averaged 120 g. for the females and 160 g. for the males. When killed 2 years later the experimental animals were found to have grown equally with the controls. The average body weights were as follows : control males 307 g., subtotally thyroidectomized females 249 g.

### *Thyroids, macroscopic findings*

The thyroid remnants were measured but not weighed. The control thyroids and the residual right lobes of the hemi-thyroidectomized rats all appeared normal. The findings in the 26 survivors of subtotal thyroidectomy were as follows : no thyroid tissue was identified in 3 rats, all females. In 10 animals, 8 females and 2 males, the remnant was very small measuring less than 2·5 mm. in its greatest diameter, i.e. less than half a lobe in volume. The remnant occupied the site of the isthmus and was attached to overlying muscle. In 1 female the isthmic remnant was missing but a small nodule of thyroid tissue was present at the site of the right upper pole. In another female the isthmic remnant was expanded by a primary thyroid tumour that infiltrated the local muscle and vessels. In 4 animals, 3 females and 1 male, the remnant was estimated to be the size of half a thyroid lobe, consisting of isthmus plus a contiguous portion of left or right lobe. In 9 animals, 3 females and 6 males, the remnant was the size of one lobe.

### *Thyroids, microscopic findings*

There was no difficulty in differentiating remnants, small or large, from control thyroids. The normal microscopic appearance of zoning due to contrast in size between the large peripheral and small central follicles (Fig. 1) was poorly marked or absent in the remnants as a result of extension of small follicles towards the periphery (Fig. 2). Colloid storage was much reduced (Fig. 3 and 4). The cells were taller (Fig. 3 and 4). Thus the histology of the remnants was that of much more active thyroid tissue than the controls. The epithelium of the remnants was heavily loaded with fine intracytoplasmic orange-brown pigment granules (Fig. 5), also present but far less prominent in controls. Strongly PAS

positive intracytoplasmic droplets were present in scattered follicle cells both of controls and remnants. Light cells (Axelrad and Leblond, 1955; Stux *et al.*, 1961), large pale epithelial cells lying singly or in small groups basally within follicles, were seen more frequently in the controls (Fig. 6). Minute nodular collections of light cells and occasional aggregates of such nodules were seen in some controls and very occasionally in thyroid remnants. Small collections of epithelial cells with hyaline cytoplasm (Fig. 7), sometimes of a signet-ring appearance, were seen in half the experimental thyroids and in only one of the controls. These cells resemble the illustration of cells in the thyroids of iodide deficient rats described by Axelrad and Leblond (1955) as granular vacuole cells.

An attempt was made to quantify the changes by measurement of follicle cell height in 50 cells per control or remnant. Zoning, colloid abundance, abundance of intracytoplasmic orange-brown granules and incidence of light cells were graded arbitarily into categories. These measurements were done without reference to remnant size. When the results were later correlated with remnant size a good inverse relationship was found between size of thyroid and histological signs of activity, Tables I and II. The inverse correlation between cell height and thyroid remnant size is shown in Table I where it is seen that the mean cell height increased from 7·2 $\mu$. in the 13 controls to 11·3 $\mu$. in the 9 rats with thyroid remnants less than half-a-lobe in size. The detailed findings were submitted to J A. Heady for statistical analysis. He found a significant difference between the means of the remnant size groups, and also that the regression, i.e. the uniform tendency for cell height to be taller with decreasing remnant size, was significant; P was less than 0·001 in both instances.

### EXPLANATION OF PLATES

FIG. 1.—Control thyroid showing large peripheral and smaller central follicles. TriPAS. ×33.

FIG. 2.—Thyroid remnant less than half a lobe in size, showing loss of zoning and gross reduction in colloid. TriPAS. ×33.

FIG. 3.—Control thyroid. Higher power of Fig. 1. TriPAS. ×230.

FIG. 4.—Thyroid remnant. Higher Power of Fig. 2. TriPAS. ×230.

FIG. 5.—Thyroid remnant showing fine intracytoplasmic pigment-granules. H. and E. ×800.

FIG. 6.—Focus in control thyroid of massive proliferation of light cells separating atrophic follicular cells from follicular basement membrane. H. and E. ×335.

FIG. 7.—Thyroid remnant showing hyalinization of follicular cells (granular vacuole cells of Axelrad and Leblond, 1955). H. and E. ×370.

FIG. 8.—Thyroid remnant containing a small follicular adenoma with crowded nuclei. H. and E. ×100.

FIG. 9.—Thyroid remnant replaced by primary carcinoma showing invasion of extra-thyroidal veins. H. and E. ×15.

FIG. 10.—Higher power of carcinoma in Fig. 9. H. and E. ×325.

FIG. 11.—Higher power of invaded veins in Fig. 9. H. and E. ×105.

FIG. 12.—Pituitary of subtotally thyroidectomized rat showing proliferation of beta cells containing prominent T granules. TriPAS. ×735.

FIG. 13.—Pituitary of control rat showing a massive chromophobe cell adenoma. H. and E. ×16.

FIG. 14.—Higher power of chromophobe cell adenoma in Fig. 13 showing crowded nuclei and non-voluminous cytoplasm and cystic spaces containing red cells. H. and E. ×500.

FIG. 15.—Thyroidectomy cell adenoma showing voluminous cytoplasm and recognizable negative Golgi images. A mitotic figure is present in the centre. H. and E. ×400.

FIG. 16.—Thyroidectomy cell adenoma showing very occasional cells with intacytoplasmic T granules. TriPAS. ×475.

FIG. 17.—Probable thyroidectomy cell adenoma showing bizarre giant nuclei and mitotic figures. H. and E. ×500.

FIG. 18.—Thyrotroph cell adenoma showing very fine beta cell granulation. TriPAS. ×760.

1

2

3

4

Doniach and Williams.

5

6

7

8

Doniach and Williams.

9

10

11

12

13

14

15

16

17

18

Doniach and Williams.

TABLE I.—*Thyroid Cell Height in Relation to Remnant Size 2 Years After Partial Thyroidectomy*

| Remnant size | Number of rats | Mean cell height in microns | Range of mean cell heights of individual rats |
|---|---|---|---|
| < ½ lobe | 9 | 11·3 | 8·7–13·4 |
| ½ lobe | 4 | 10·8 | 10·2–11·6 |
| 1 lobe | 11 | 9·2 | 7·9–12·6 |
| Controls | 13 | 7·2 | 5·7–9·6 |

TABLE II.—*Thyroid Histology in Relation to Remnant Size*

| Number of rats | Remnant size | Colloid abundance* | | | Abundance of pigment* | | | Abundance of light cells* | | | Tumours | |
|---|---|---|---|---|---|---|---|---|---|---|---|---|
| | | + | ++ | +++ | 0 | ++ | +++ | 0 | ++ | +++ | Adenomas | Carcinomas |
| 13 | ½ lobe or less | 11 | 2 | 0 | 0 | 0 | 13 | 9 | 4 | 0 | 2 | 1 |
| 11 | 1 lobe | 0 | 8 | 3 | 0 | 2 | 9 | 3 | 5 | 3 | 0 | 0 |
| 13 | Controls | 0 | 0 | 13 | 3 | 8 | 2 | 0 | 4 | 9 | 0 | 0 |

* Number of rats per category.

Sections of thyroid were lost from 2 experimental males with lobe sized remnants, and from 1 female with a remnant of less than half a lobe in size.

*Thyroid tumours*

One adenoma, 0·3 mm. in diameter, was found in a remnant of smallest size in a female rat and one, 0·4 mm. in diameter, in a remnant of half lobe size in a male rat. The adenomas were similar in appearance : follicular with colloid formation, the epithelium basophilic with crowded overlapping nuclei (Fig. 8). The tumour cells were mostly devoid of orange-brown pigment granules in contrast to the surrounding non-neoplastic epithelium which contained pigment in abundance. The adenoma in the male rat showed occasional mitoses. In one female rat the tiny remnant was expanded and infiltrated by a primary tumour 2 × 2 × 1 mm. Sections show a mixed follicular and solid carcinoma with striking permeation of large extra-thyroidal veins by tumour (Fig. 9 and 11). The solid areas consist of massed follicular cells, poor in cytoplasm, arranged in alveoli some of which contain a little central colloid (Fig. 10) ; mitoses are present in moderate number.

*Pituitary histology*

Pituitary sections were available from all 13 controls and from 29 of the 30 experimentals. Well granulated alpha cells were present in equal number in the experimentals and controls. Identification of thyrotroph (beta) cells by their central position within each lobe, large size, angular shape, strongly positive PAS and aldehyde-fuchsin staining of their granules (Purves and Griesbach, 1956) proved straightforward. Such cells were plentiful in the controls. They were markedly reduced in the experimentals which showed the changes typical of thyroidectomy (Purves and Griesbach, 1956) : degranulation and increase in size and number of beta cells many of which contain coarse strongly PAS positive granules, " T " granules or droplets (Catchpole, 1949 ; Pearse, 1952) (Fig. 12). As a result of the degranulation, the overall PAS staining was reduced. The enlarged beta cells were often grouped in small clumps and showed occasional mitotic figures and giant bizarre nuclei. The classical hyaline vesicles in beta cells observed by Purves and Griesbach (1956) soon after total thyroidectomy were present in small numbers only.

We found a definite correlation between intensity of thyroidectomy changes in the pituitary histology and smallness of size of thyroid remnant. T granules were seen in over half the experimentals, being especially numerous in the pituitaries of animals with the smallest thyroid remnant (present in 11 out of 12 rats). A few T granules were seen in 2 and none in 11 of the 13 controls. Mitoses were seen in 2 of the 13 controls, in 6 of the 12 rats with a small thyroid remnant and in 4 of the remaining 17 animals. These findings refer to the non-adenomatous areas of pars anterior. Finally, well granulated thyrotrophs were numerous in 12 of the 13 controls, in only 1 of the 12 rats with a small thyroid remnant and in 7 of the remaining 17 animals.

*Pituitary tumours*

Tumours were recognized as cell aggregates arranged in foci easily distinguished from surrounding pars anterior tissue by their greater uniformity and their tendency to compress surrounding cells (Fig. 13). Adenomas derived from beta cells (thyrotrophs) vary in cytology. Those containing cells resembling the granulated beta cells of normal glands we have called thyrotroph cell adenomas. Those made up of large chromophobe degranulated cells showing occasional T granules as seen after thyroidectomy we have called thyroidectomy cell adenomas.

*Controls.*—No pituitary tumours were found in the 3 males. They were present however in 6 of the 10 females. Three pituitaries contained single tumours, 2 contained two and 1 contained three tumours. All were chromophobe cell adenomas. They varied in diameter from 0·3 to 3·0 mm. and were made up of small or medium sized cells containing little or moderate amounts of cytoplasm. In some tumours the nuclei were small, in others medium sized with scattered large types. Mitoses were seen rarely. Very occasional alpha cells and mucoid (basophil) cells were identified within the tumours. The adenomas were vascular, often haemorrhagic and many contained cystic spaces sometimes filled with red cells (Fig 14). Though clearly demarcated, the tumours were not encapsulated. One large adenoma infiltrated the contiguous pars intermedia.

*Experimentals*

(1) *Rats whose thyroid remnants were less than half a lobe in size* —Pituitary tumours were present in 11 of these 12 animals, and were considered in 6 animals to be of beta cell origin. Four females showed thyroidectomy cell adenomas, moderately well demarcated, not encapsulated, averaging 0·7 mm. in diameter. They were made up of nests and trabeculae of large polyhedral cells with voluminous cytoplasm often showing a large negative Golgi image but no beta granulations (Fig. 15). Scattered throughout were occasional cells containing T granules (Fig. 16). Mitoses were present in moderate number as well as a few giant bizarre nuclei (Fig. 15). One further female showed a tumour of similar appearance except that no T granules were identifiable (Fig. 17) ; this was classified as a probable thyroidectomy cell adenoma. The pituitary of another female rat was enlarged by a striking widespread proliferation of thyroidectomy cells loaded with

T granules (Fig. 12), arranged in clumps and trabeculae with frequent mitoses but not demarcated into nodules. This was regarded as pre-adenomatous thyroidectomy cell hyperplasia. One female rat pituitary showed an adenoma 1·5 mm. in diameter made up of large polyhedral cells many of which contained fine granules that were PAS positive, aldehyde-fuchsin positive and basophil in Heidenhain's azan trichrome stain, typical beta granulation of thyrotroph cells (Fig. 18), classified as a thyrotroph cell adenoma.

Three female rats showed chromophobe cell adenomas varying from 0·7 to 2·75 mm., single tumours in 2 animals, double in 1. Mitoses were quite frequent, infiltration of the pars intermedia was noted in 3 of the 4 tumours. In each of two rats, both males, there was a single minute gonadotroph cell adenoma 0·15 to 0·2 mm. in diameter. The cells were ovoid and filled with fine PAS positive, aldehyde-fuchsin negative, basophilic granules. The negative Golgi image was prominent.

(2) *Rats whose thyroid remnant was half a lobe in size.*—Of these 4 rats, 1 female and 1 male showed no tumour. There was a single pituitary tumour in each of the remaining 2 females: a large 1·3 mm. probable thyroidectomy cell adenoma and a large 1·6 mm. chromophobe cell adenoma.

(3) *Rats whose thyroid remnant was 1 lobe in size.*—Of these 13 rats, 6 were males, 3 of whom showed pituitary tumours: 2 small 0·3 mm. gonadotroph cell adenomas and 1 very large probable thyroidectomy cell adenoma 2·5 mm. in diameter, unusually rich in mitoses and bizarre giant nuclei, infiltrating flanking pars anterior tissue. Of the remaining 7 females, 3 showed tumours: 1 thyrotroph cell adenoma 0·4 mm. in diameter, 1 probable thyroidectomy cell adenoma 0·5 mm. in diameter and 1 chromophobe cell adenoma 0·9 mm. in diameter.

TABLE III.—*Incidence of Pituitary Tumours*

| Size of thyroid remnant | Total number of rats | Number of rats with beta cell tumours | | | Number of rats with non-beta cell tumours | | Total number of rats with pituitary tumours (number with beta cell tumours in brackets) |
|---|---|---|---|---|---|---|---|
| | | Thyrotroph cell adenomas | Thyroidectomy cell adenomas | Probable thyroidectomy cell adenomas | Chromophobe cell adenomas | Gonadotroph cell adenomas | |
| ½ lobe or less | 16 | 1 | 4 | 2 | 4 | 2 | 13 (7) |
| 1 lobe | 13 | 1 | Nil | 2 | 1 | 2 | 6 (3) |
| Controls | 13 | Nil | Nil | Nil | 6 | Nil | 6 (0) |

The above findings are summarized in Table III. The correlation of thyroid remnant tumours with pituitary tumours was as follows: the pituitary of the rat with the primary thyroid carcinoma showed the pre-adenomatous thyroidectomy cell hyperplasia described above, consistent with unusually excessive secretion of TSH. One thyroid remnant adenoma was associated with a probable thyroidectomy cell adenoma. The pituitary associated with the other thyroid remnant adenoma was free of tumour and showed moderate T granulation. The thyroid remnant of the male rat in the last group associated with the malignant looking probable thyroidectomy cell adenoma did not show morphological signs of activity in excess of the other thyroid remnants in the group.

### DISCUSSION

In the present experiment, subtotal thyroidectomy led to a still greater increase in follicular cell height than that noted by Logothetopoulos and Doniach (1955) after hemi- and three-quarter thyroidectomy. The hypertrophy was maintained for 2 years, the major part of the rat's life-span, associated with histological evidence in the pituitary of increased TSH secretion. A similar picture of activity was noted by Dent *et al.* (1955) in thyroid remnants in mice many months after near total thyroidectomy and was recently recorded in the thyroid remnants of hemi-thyroidectomized cats (Knigge, 1961). Ingle and Cragg (1939) described the unexpected maintenance of morphological activity of thyroid autotransplants for 3 months in completely thyroidectomized rats. Thus, evidence of the maintenance of a raised TSH after partial thyroidectomy has also been observed by others and in other species.

We do not know if thyroid function was restored to normal in the present experiments, nor why the thyroid remnants varied so much in size 2 years after operation. The normal growth of the animals implies considerable restoration of thyroid function. There was a good inverse correlation between remnant size, thyroid follicular cell height and histological evidence in the pituitary of TSH secretion. The variation in final sizes of remnants might well reflect variation in the proportion of thyroid gland removed initially. The larger the proportion of thyroid removed at operation the greater would be the resultant fall in thyroxine synthesis and consequently the greater stimulus to TSH secretion. Once the initial hyperplasia and hypertrophy of the remnant follicular cells achieves a sufficient iodine turnover to maintain a normal daily output of thyroid hormone, with an accompanying reduction in colloid store, there does not appear to be any intrinsic tendency to alter the situation. The rat appears physiologically content to derive its thyroxine from a smaller thyroid gland than normal stimulated by a higher level of TSH. An alternative possibility is that subtotal thyroidectomy inflicts too great a challenge for complete restoration of thyroid function. If so, this is unlikely to be due simply to limitation of growth capacity of the thyroid remnant since the thyroid gland is capable of undergoing a ten to twenty-fold increase in mass under the influence of goitrogens. However, without resolving these problems, the findings suggest that the rise in TSH secretion stimulated by partial thyroidectomy is perpetuated in rats and is proportional to the fraction of thyroid gland removed.

The effects of old age and of prolonged excess TSH stimulation, induced by an iodine deficient diet, on the histology of the rat thyroid have been admirably described by Axelrad and Leblond (1955). Our findings were essentially similar to theirs. We agree with their interpretation that the intracytoplasmic orange-brown granules in the follicular cells are probably wear and tear pigment (Fig. 5). The greater intensity of granules in follicular cells of the smaller more active remnants and their virtual absence from the neoplastic thyroid cells point to their reflection of long sustained heightened secretory activity. We observed the light cells (Fig. 6) described by Axelrad and Leblond, but in contrast to their findings, we found light cell hyperplasia and nodule formation in greater frequency in controls than in hyperplastic glands. Hyaline thyroid cells (Fig. 7) (granular vacuole cells of Axelrad and Leblond) were present in larger aggregates and more frequently in our experimental than control rats. Axelrad and Leblond (1955) observed their development in the active thyroids of iodine deficient rats. It is interesting to note that their development has not been recorded in goitrogen treated rats. Though adenoma formation was not marked in our experimental rats, the development of an indubitable thyroid carcinoma confirms the carcinogenic action on the thyroid of prolonged excess TSH stimulation. This action occurred without the aid of carcinogenic or goitrogenic chemicals or radiation and is thereby comparable with the demonstration by Axelrad and Leblond (1955) and by Bielschowsky (1953) of the carcinogenic action in rats of a low iodine diet.

The pituitary histology proved interesting. The presence of abundant well granulated alpha cells in the partially thyroidectomized rats rules out gross thyroxine deficiency. Together with the normal development and growth in body weight these findings fit, though do not prove, the possibility that compensatory hypertrophy of the thyroid remnant had been sufficient to restore thyroxine secretion to normal. We did not measure thyroid $^{131}I$ uptake in these animals. In rats maintained on a diet deficient in iodine, Axelrad and Leblond (1955) noted that alpha cells were scanty and poorly stained. This is typical of gross thyroxine deficiency in rats (Griesbach and Purves, 1945). The pituitaries were more enlarged than in our experiment and many were noted to contain grossly visible tumours. Thus, our animals must have been nearer to physiological normality with regard to blood thyroxine levels and suffered no lack of growth hormone.

The widespread proliferation of thyroidectomy cells in the pituitaries of our partially thyroidectomized rats is typical of heightened TSH secretion (Griesbach and Purves, 1945). At this stage, 2 years after operation, hyaline vacuoles were sparse, T granules numerous. The presence of occasional mitoses in thyroidectomy cells could be regarded as evidence of continuation of the stimulus to excess TSH secretion.

Both spontaneous anterior pituitary tumours (Wolfe, Brian and Wright, 1938) and induced functioning tumours have been described in the rat (reviewed by Bielschowsky and Horning, 1958). Identification of the cell type of very actively functioning adenomas can be difficult or impossible since the type granules tend to disappear from actively secreting cells. Chromophobe cells may be either undifferentiated precursor cells or else degranulated active secretors. The latter usually show a larger nucleus, more voluminous cytoplasm and often a large negative image of the Golgi apparatus. Furthermore, in tumours, the chromophobe state may sometimes represent a loss of function through anaplasia. The loss of granulation of functioning beta cell tumours in radiothyroidectomized mice was noted and discussed by Halmi and Gude in 1954. Furth and Clinton (1958) found numerous fine ovoid granules regarded as secretory in nature in electron microscopy of a mouse functioning thyrotroph cell tumour that was chromophobe in light microscopy.

We were able to identify 6 adenomas of beta cell origin in our experimental animals. No beta cell tumours were found in the controls (Table III). Thus the stimulus of partial thyroidectomy is equivalent to thyroxine deficiency in its provocation of pituitary beta cell hyperplasia and eventual neoplasia. The induction of such tumours by ablation of thyroid function has been demonstrated, mostly in mice, by a number of workers (Halmi and Gude, 1954 ; Furth and Clinton, 1958 ; Israel and Ellis, 1961). Moreover, Goldberg and Chaikoff (1951) and Gorbman (1952) showed that their development in radiothyroidectomized mice was prevented by maintained administration of thyroxine.

The infiltration of local tissue by some of our chromophobe adenomas has been observed in similar tumours in the human pituitary (Russell, 1961). There were 6 chromophobe cell adenomas in the 13 contols and 5 in the 29 experimentals (Table III). Mitoses were abundant in the latter. Griesbach and Purves (1960) classified similar spontaneously occurring tumours as acidophil-chromophobes, thought to be degranulated or lightly granulated as a result of secretory activity. The poverty of cytoplasm in the cells of the chromophobe adenomas in our rats suggested a lack of secretory activity. The possibility might be considered that some of the chromophobe tumours seen frequently in elderly rats are made up of undifferentiated " mother-cells " that are capable of differentiation to specialized secretory cells of a type depending on the stimulus to which the pituitary is submitted. Thus, after thyroidectomy a few tumours might arise from beta cell differentiation of chromophobe adenomas though the majority probably develop from established beta cells. This hypothesis would account for the reduced number of chromophobe adenomas in our experimental rats compared with the controls.

In conclusion, we have found that the single surgical manoeuvre of subtotal thyroidectomy in the rat may prove both carcinogenic to the thyroid remnant and lead to the development of beta adenomas in the pituitary gland.

### SUMMARY

Twenty-six rats surviving subtotal thyroidectomy at the age of 8 weeks, 4 surviving hemi-thyroidectomy and 13 controls were killed two years after operation. The thyroid remnants were considerably smaller than the thyroids of the controls, showed a histological picture of far greater activity and, in addition, 2 adenomas and 1 primary thyroid carcinoma.

Histology of the pituitary glands showed well marked beta cell proliferation and thyroidectomy cell changes in the experimental animals together with 4 thyroidectomy cell adenomas, 2 thyrotroph cell adenomas and 4 probable thyroidectomy cell adenomas. No beta cell adenomas were identified in the control pituitaries. Chromophobe cell adenomas were present in both experimentals and controls.

The experimental animals showed normal body development, weight gain and pituitary alpha cell granulation, thus implying that the thyroid and pituitary compensatory hyperplasia had produced considerable restoration of thyroid function.

We are grateful to J. A. Heady, of the M.R.C. Department of Social Medicine, the London Hospital Medical College for the statistical analysis; to Mrs. Susan Hussey for technical assistance, to John King for the photomicrographs and to the British Empire Cancer Campaign for financial support.

### REFERENCES

AXELRAD, A. A. AND LEBLOND, C. P.—(1955) *Cancer*, **8**, 339.
BIELSCHOWSKY, F.—(1949) *Brit. J. Cancer*, **3**, 547.—(1953) *Ibid.*, **7**, 358.
*Idem* AND HORNING, E. S.—(1958) *Brit. med. Bull.*, **14**, 106.
CATCHPOLE, J.—(1949) *J. Endocrin.*, **6**, 218.
DENT, J. N., GADSDEN, E. L. AND FURTH, J.—(1955) *Cancer Res.*, **15**, 70.

FURTH, J. AND CLINTON, K. H.—(1958) *Ciba Foundation Colloquia in Endocrinology*, **12**, 3. London (J. & A. Churchill).
GOLDBERG, R. L. AND CHAIKOFF, I. L.—(1951) *Endocrinology*, **48**, 1.
GORBMAN, A.—(1952) *Proc. Soc. exp. Biol., N.Y.*, **80**, 538.
GRIESBACH, W. E. AND PURVES, H. D.—(1945) *Brit. J. exp. Path.*, **26**, 13.—(1960) *Brit. J. Cancer*, **14**, 49.
*Idem*, KENNEDY, T. H. AND PURVES, H. D.—(1945) *Brit. J. exp. Path.*, **26**, 18.
HALL, W. M.—(1955) unpublished results quoted by BIELSCHOWSKY, F. (1955) *Brit. J. Cancer*, **9**, 86.
HALMI, N. S.—(1952) *Stain. Tech.*, **27**, 61.
*Idem* AND GUDE, W. D.—(1954) *Amer. J. Path.*, **30**, 403.
INGLE, D. J. AND CRAGG, R. W.—(1939) *Endocrinology*, **24**, 550.
ISRAEL, M. S. AND ELLIS, R. I.—(1961) *Brit. J. Cancer*, **15**, 763.
KNIGGE, K. M.—(1961) *Anat. Rec.*, **141**, 151.
LOGOTHETOPOULOS, J. H. AND DONIACH, I.—(1955) *Brit. J. exp. Path.*, **36**, 617.
PEARSE, A. G. E.—(1949) *J. Path. Bact.*, **61**, 195.—(1952) *Ibid.*, **64**, 791.
PURVES, H. D. AND GRIESBACH, W. M.—(1947) *Brit. J. exp. Path.*, **28**, 46.—(1951) *Endocrinology*, **49**, 244 and 652.—(1956) *J. Endocrin.*, **13**, 365.
RUSSELL, D. S.—(1961) in: W. A. D. ANDERSON'S "Pathology" 4th edition, 1961, St. Louis, U.S.A. (C. V. Mosby Co.) p. 994.
STUX, M., THOMPSON, B., ISLER, M. AND LEBLOND, C. P.—(1961) *Endocrinology*, **68**, 292.
WOLFE, J. M., BRIAN, W. R. AND WRIGHT, A. W.—(1938) *Amer. J. Cancer*, **34**, 352.